# A Versatile and Open-Source Rapid LED Switching System for One-Photon Imaging and Photo-Activation

**DOI:** 10.3389/fncel.2018.00530

**Published:** 2019-01-17

**Authors:** Arne Battefeld, Marko A. Popovic, Dirk van der Werf, Maarten H. P. Kole

**Affiliations:** ^1^Department of Axonal Signaling, Netherlands Institute for Neuroscience, Royal Netherlands Academy of Arts and Sciences, Amsterdam, Netherlands; ^2^Grass Laboratory, Marine Biological Laboratory, Woods Holem, MA, United States; ^3^Department of Mechatronics, Netherlands Institute for Neuroscience, Royal Netherlands Academy of Arts and Sciences, Amsterdam, Netherlands; ^4^Cell Biology, Faculty of Science, Utrecht University, Utrecht, Netherlands

**Keywords:** Arduino, μManager, microscopy, LED, high-speed imaging, Propeller, calcium imaging

## Abstract

Combining fluorescence and transmitted light sources for microscopy is an invaluable method in cellular neuroscience to probe the molecular and cellular mechanisms of cells. This approach enables the targeted recording from fluorescent reporter protein expressing neurons or glial cells in brain slices and fluorescence-assisted electrophysiological recordings from subcellular structures. However, the existing tools to mix multiple light sources in one-photon microscopy are limited. Here, we present the development of several microcontroller devices that provide temporal and intensity control of light emitting diodes (LEDs) for computer controlled microscopy illumination. We interfaced one microcontroller with μManager for rapid and dynamic overlay of transmitted and fluorescent images. Moreover, on the basis of this illumination system we implemented an electronic circuit to combine two pulsed LED light sources for fast (up to 1 kHz) ratiometric calcium (Ca^2+^) imaging. This microcontroller enabled the calibration of intracellular Ca^2+^ concentration and furthermore the combination of Ca^2+^ imaging with optogenetic activation. The devices are based on affordable components and open-source hardware and software. Integration into existing bright-field microscope systems will take ∼1 day. The microcontroller based LED imaging substantially advances conventional illumination methods by limiting light exposure and adding versatility and speed.

## Introduction

The continuous improvement of equipment and methodologies to visualize cells and their fine processes is critical to the advancement of neuroscience research. Cellular membranes are phase objects and with the many layers in intact brain tissue the identification of individual cells and their subcellular processes provides a major challenge. The application of long infrared (IR) wavelengths for visualization of cells in brain tissue reduces light scattering and in combination with different contrast enhancing methods, neuronal processes can be directly visualized (Dodt and Zieglgänsberger, 1990; Stuart et al., 1993; Dodt et al., 1999). Loading unlabeled neurons with chemical fluorescent dyes through the patch pipette further facilitated visualization of processes for targeted subcellular recording (Stuart et al., 1993). More recently, genetic strategies for fluorescence reporter expression (Zhuo et al., 1997; Feng et al., 2000; Mallon et al., 2002) improved experimental targeting of specific cell classes.

Many of the above mentioned approaches benefit from the combination of transmitted light with fluorescence light, but existing hardware solutions have pros and cons. Two-photon or confocal spinning disk microscopy are both ideal to simultaneously monitor subcellular structures and patch pipettes, exert minimal phototoxicity and provide sufficient spatial resolution to identify small diameter structures such as fine dendrites and axons (Nevian et al., 2007; Sasaki et al., 2012; Kole and Popovic, 2016). However, in comparison to one-photon microscopes the equipment is expensive. While conventional one-photon epifluorescence microscopy setups are more affordable they suffer from poor control of the excitation light. For example, conventional arc lamps have a very high luminance output and several emission peaks that are useful for the imaging of various fluorescence proteins. Adjustment of light intensity has to be achieved with neutral density filters. Short exposure times that limit phototoxicity (Icha et al., 2017) have to be established by third party shutters adding cost and potential sources of vibration. Furthermore, the widespread use of IR differential interference contrast (DIC), to improve the contrast of *in vitro* brain slices, prohibits the simultaneous use of transmitted and fluorescence light and requires switching of the analyzer and fluorescence cube. Alternatives for DIC are either the simpler oblique contrast or the Dodt gradient contrast illumination (Dodt et al., 1999) both enabling fluorescence illumination without switching components in the light path.

In recent years, high-power light emitting diodes (LEDs) from commercial suppliers or purpose built solutions (Albeanu et al., 2008; Rösner et al., 2013; Bosse et al., 2015) found their way into microscope illumination systems. High-power LEDs provide a low-noise and high intensity light source for microscopy. Combining LEDs for dual excitation fluorescence allows microsecond rapid switching between excitation wavelengths. Rapid LED switching has previously been shown to allow pseudo-simultaneous imaging of two fluorescence reporters at high speed (Miyazaki and Ross, 2015). However, the possibilities to implement fast pseudo-simultaneous imaging is often limited by the specific equipment. Similarly, a simple system that integrates fluorescence assisted patch-clamping into existing microscope setups is currently lacking.

Here, we present a number of simple and cost-effective solutions based on open-source hardware and LED illumination that can be easily adapted to most microscopes. We created a GitHub repository with instructions, code and circuit diagrams to build these microcontroller devices. First, we show a device that allows the near-continuous overlay of fluorescence and bright-field images for fluorescence assisted patching. Second, we present an extended solution for combining photo-activation and Ca^2+^ imaging. Third, we demonstrate the possibility to perform ratiometric imaging at microseconds speed. The combination of open-source hardware and software provides the backbone for an affordable and versatile system advancing epifluorescence microscopy setups for optical and electrophysiological recordings.

## Methods

### Animals, Slice Preparation and Whole-Cell Patch Clamp Recordings

This study was carried out in accordance with the recommendations of EU directive 2010/63/EU, Dutch law and the institutional IACUC committee. The protocols involving animal work were approved by the local ethics committees (IACUC or KNAW-DEC). Male Wistar rats at an age between 3 to 7 weeks (Charles River and Janvier) were kept on a 14/10 or 12/12 light/dark cycle. Mice were kept on a 12/12 light/dark cycle. All animals had unrestricted access to food and were fed a standard diet. The following mouse lines were used: R26-CAG-LSL-2XChETA-tdTomato (Jax: #017455) x Rbp4-cre_KL100Gsat/Mmucd (RRID:MMRRC_031125-UCD); PLP-CFP (Hirrlinger et al., 2005; Battefeld et al., 2016). Coronal and parasagittal brain slices (300 μm) were made as described in detail previously (Davie et al., 2006; Battefeld et al., 2016). Whole-cell patch clamp recordings were performed as described previously (Battefeld et al., 2016) and controlled by either AxoGraph X (RRID:SCR_014284, AxoGraph Scientific, Sydney, NSW, Australia) or pClamp (RRID:SCR_011323, Molecular Devices) software.

### Microscope Configuration, Filters and Cameras

For combining fluorescence and transmitted light illumination we used an optical light path that allowed simultaneous imaging without the need for changing fluorescence filter cubes. This prerequisite was fulfilled by either the Dodt gradient contrast (Dodt et al., 1999) or oblique condenser illumination. We tested the equipment on an Examiner A1 (Zeiss, Thornwood, NY, United States) with built in Dodt gradient contrast or an BX51WI (Olympus, Leiderdorp, Netherlands) with an oblique illumination condenser (WI-OBCD, N.A. 0.8). For simultaneous imaging of fluorescence and transmitted light, filter cubes were equipped with an excitation filter 530/30 nm (Chroma, Bellows Falls, VT, United States), a dichroic mirror 550lpxr (Chroma) and a long pass filter 570 nm (FGL570, Thorlabs). We successfully utilized alternative combinations of LED and filtercubes for CFP (U-MCFPHQ, Olympus), GFP (U-MWB, Olympus), and RFP (U-MWG2, Olympus). These standard filter cubes often required replacement of the emission filter with a long-pass emission filter. A sufficient and inexpensive solution is to use a long-pass colored glass filter (from Schott corporation) for the desired cut-on wavelength. The long-pass filter allows the combination of fluorescence with near infra-red light used for the transmitted light. For simultaneous Ca^2+^ imaging and optogenetic activation two fluorescence light paths were arranged on top of each other. This configuration allows independent control of field illumination for Ca^2+^ indicator and optogenetic light paths. Ideally, the field stop opening is kept small for Ca^2+^ imaging to reduce bleaching and phototoxicity and large (fully open) to increase the activation efficiency of light-gated channelrhodopsins. The lower fluorescence light path was routed through the standard fluorescence illuminator (BX-RFA, Olympus). A U-DP coupled to U-DP1XC was mounted on top the BX-RFA. Olympus microscopes that we used for patching and fast imaging were equipped with a dual camera intermediate attachment (U-TRU, Olympus) allowing the connection of two cameras. We have successfully utilized CCD and scientific CMOS (sCMOS) based cameras for fluorescence assisted patching that include Xyla 4.2 (Andor, Concord, MA, United States), CoolSnap EZ (Photometrics, Tucson, AZ, United States), CoolSnap ES2 (Photometrics), and Evolution QEi (QImaging, Surrey, BC, Canada).

### LEDs and LED Driver Hardware

For epifluorescence illumination, we used various combinations of collimated LEDs with peak wavelengths of 340, 420, and 530 nm (all from Thorlabs, Newton, NJ, United States). In addition, we used custom built LEDs with peak wavelengths of 448 nm (SP-01-V4, Luxeonstar) and 590 nm (SP-01-A8, Luxeonstar). As transmitted light source, we used a collimated infrared 730 nm LED (M730L4, Thorlabs) mounted into the transmitted light port. Besides LEDs with single peak wavelengths we successfully used a high-power white LED (LZ1-00CW02, LED Engin Inc., San Jose, CA, United States) that allowed rapid and easy switching between different fluorescence excitation wavelengths without requiring several LEDs for each excitation wavelength. Custom built LEDs were mounted on a heatsink, collimated and arranged into the light path via a cage system similarly as previously described (Albeanu et al., 2008; Bosse et al., 2015). LEDs were equipped with proper cooling (passive heat sinks) and were not driven continuously to increase their lifetime. LEDs were TTL pulse controlled and LED drivers (LEDD1B, Thorlabs) allowed manual adjustment for light intensity in *continuous wave* or *trigger* mode or analog intensity control in *analog modulation* mode. We successfully tested the Cyclops LED driver providing even faster current delivery to LEDs and allowing optical feedback to achieve improved LED intensity stability (open-ephys project, Newman et al., 2015). An application example summarizing utilized LEDs and drivers can be found in Supplementary Table 1. As an alternative to LED light sources, other light sources (arc-lamp or laser) in combination with pre-existing mechanical shutters could be used, thereby reducing the need for new hardware. In this case, the TTL pulses trigger opening and closing of shutters or gating of lasers. We successfully used this approach to trigger mechanical shutters of Vincent Associates Inc. (Rochester, NY, United States) or laser light sources (89 North, Williston, VT, United States).

### Single-Cell Ca^2+^ Imaging

For Ca^2+^ imaging experiments, we supplemented the intracellular solution with either 200 μM Cal-590 potassium salt (20518, AAT Bioquest, Sunnyvale, CA, United States) or 200 μM fura-2 pentapotassium salt (F1200, Thermo Fisher Scientific). Cal-590 was excited with a collimated 590 nm LED (Luxeonstar) passed through an excitation filter 538/84 (FF01-538/84-25, Semrock, Rochester, NY, United States) reflected by a 580 nm beam splitter (FF580-FDi01-25x36, Semrock) toward the sample and emission filtered with a 593 nm long-pass filter (FF01-593/LP-25, Semrock). Channelrhodopsin activation combined with Ca^2+^ imaging was performed in juvenile animals (P14 to P20). The channelrhodopsin variant ChETA was activated with a non-optimal excitation wavelength of 448 nm resulting in reduced photo-activation in response to blue activation light. We could not detect optical crosstalk between photo-activation light and the excitation light used for imaging of the red shifted Ca^2+^ indicator Cal-590.

Fura-2 was alternately excited with 340 and 420 nm LEDs that were collimated with a UV-optimized aspheric lens for 340 nm (#33-953, Edmund Optics, York, United Kingdom) and a 1^′′^ plano-convex lens (N-BK7, Thorlabs) for 420 nm. The 420 nm LED was passed through a 420/10 (FF01-420/10-25, Semrock) excitation filter and the 340 nm LED through a 340/22 nm excitation filter (FF01-340/22-25, Semrock) before combining the two light paths by passing/reflecting with a 405 nm dichroic mirror (Di02-R405, Semrock). We replaced the tube lens of U-DPXC1 with a UV passing plano-convex lens (#48-289, Edmund Optics) and placed a round field stop at a focal length of 17.5 cm. Light was reflected by a dichroic mirror (FF458-Di02, Semrock) through a 60× 1.0 NA water immersion objective (LUMPLFLN 60× W, Olympus) to the sample. Emission light was long-pass filtered with a cut-on wavelength of 455 nm. Exciting fura-2 at 420 nm reduced its dynamic range, but still allowed calibration.

For Ca^2+^ imaging we utilized a low resolution (80 × 80 pixel), but highly sensitive NeuroCCD camera (RedShirt Imaging, Decatur, GA, United States) mounted on the front port of U-TRU. This camera was controlled by Neuroplex software (RedShirt Imaging) and imaging time series were recorded in Neuroplex (.da file format). *Post hoc* separation of imaging channels was performed by a script available on github^1^.

### Software

We used the Arduino IDE (Arduino CC) with the Teensyduino plugins installed ^2^ to program Teensy. PropellerIDE software (Parallax) was used to program the microcontroller for POPSAR. We used μManager (RRID:SCR_000415) (Edelstein et al., 2014) for integrating microscope illumination and camera control with TEAMSTER. RedShirt Imaging NeuroCCD cameras were controlled by Neuroplex software (RRID:SCR_016193, v. 10.1.2 and v 10.2.1, RedShirt Imaging) and image acquisition trigger signals were detected by TEAMSTER and SLIDER.

### Code and Circuit Schematics

We have created an online repository on GitHub^3^ which includes all code and circuit schematics described here. Instructions and part lists for building TEAMSTER, POPSAR, and SLIDER are provided.

## Results

**FIGURE 1:**
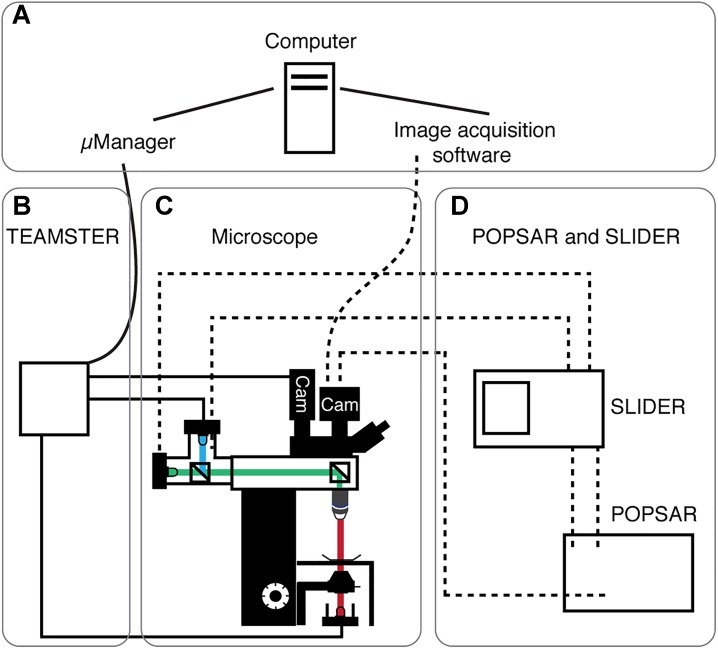
System overview of presented microcontrollers. Schematic overview showing the four main modules of the system. **(A)** Computer running μManager and/or specialized image acquisition software. **(B)** The microscope equipped with transmitted light and fluorescence LEDs. Light paths indicated (fluorescence: blue, green; transmitted: red) as well as cameras (Cam). **(C)** TEAMSTER, a microcontroller based and μManager integrated LED timing and switching unit for, e.g., patch-clamp applications (solid lines, see Figure 2). **(D)** POPSAR and SLIDER microcontroller for precise LED timing and setting of LED intensities (dashed lines) for, e.g., Ca^2+^ imaging applications (see Figures 3, 4).

Here we present three microcontroller devices that are used for controlling LEDs to provide epifluorescence and transmitted light illumination for microscopes. For targeted patch-clamp recordings from subcellular structures we designed a device that we call TEAMSTER (Teensy Micromanager based timing switch controller). This Arduino based solution can control continuous overlay of transmitted and epifluorescence LEDs on conventional microscopy patch-clamp setups (Figures 1A–C). We further adapted the illumination system and designed a device we call POPSAR (Propeller based activity generator). This microcontroller solution allows high-speed (tested up to 1 kHz) imaging with dual excitation LEDs for, e.g., fura-2 based ratiometric imaging on a single camera (Figure 1D). To achieve reproducible LED activation currents, we interconnected POPSAR with a device we call SLIDER (Simple light digitizer), which we used to precisely control current intensities for LED drivers (Figure 1D).

### TEAMSTER, a Microcontroller With μManager Integration for Fluorescence-Assisted Patch-Clamp Recordings

As basis of the LED controller device (Figure 1B) we utilized an Arduino compatible Teensy microcontroller board (v3.2) that is interfaced with μManager (Supplementary Figure 1A). TEAMSTER reads frame exposure signals from the camera to subsequently trigger illumination LEDs. A manual (force) switch was implemented to manually test LED activation. For easier integration into experimental patch-clamp setups we added a foot-switch for controlling fluorescence by user input, which helps to limit fluorescence exposure. TEAMSTER controls the LED pulse durations of transmitted and fluorescence LEDs. By keeping activation times of the transmitted light LED short (25 ms) motion blur that can occur when focusing through a sample is avoided. Once triggered, the fluorescence LED is activated for the full defined frame duration. TEAMSTER is an affordable hardware interface (Supplementary Table 2) between the μManager software to control two LED drivers via TTL outputs based on pre-programmed and user selectable modes.

**FIGURE 2:**
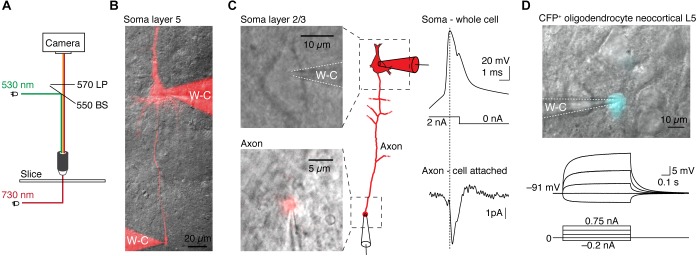
Application examples of TEAMSTER, a microcontroller for simultaneous imaging of transmitted and fluorescence light for targeted patch-clamp recordings. **(A)** Schematic showing the light paths for the combination of LEDs for fluorescence (green) and near infrared illumination (red). A long-pass filter allows passing of transmitted and fluorescence light while blocking fluorescence excitation light. **(B)** Example of a layer 5 neuron filled with Alexa dye to visualize the axon and perform a double whole-cell recording from the soma and axon bleb (not shown here). **(C)** Example images of a layer 2/3 neuron soma (top) and a corresponding axon bleb (bottom) identified after loading the neuron with Alexa 555 dye during whole-cell access. μManager was utilized to display a continuous overlay of the bright field image with Alexa 555 fluorescence allowing a cell-attached recording of the axon bleb, shown in the example traces. **(D)** Example of a targeted patch-clamp recording in a transgenic reporter mouse in which oligodendrocytes express CFP (top). Corresponding current-clamp recording of the targeted oligodendrocyte (middle, bottom).

The effectiveness of integrating TEAMSTER into patch-clamp experiments is demonstrated by two examples (Figure 2). We used a green LED (530 nm) for epifluorescence excitation and a near-IR LED (730 nm) for transmitted light illumination with either oblique illumination contrast or Dodt contrast. In combination with a long-pass emission filter, fluorescence and transmitted images can then be projected on the same camera. Timing is provided by controlling LEDs via programmed and user selectable modes in μManager. We tested the functionality by targeting cut axon blebs (Shu et al., 2006; Kole et al., 2007) from pyramidal neurons in layer 2/3 and 5, which were filled with Alexa dyes during somatic whole-cell recordings (Kole and Popovic, 2016). After ∼30 min neurons were sufficiently filled and the axon bleb could be clearly identified and targeted for electrical recording (Figures 2B,C). To live overlay the epifluorescence image of the Alexa dye with a transmitted light image we ran an additional script in μManager. The script assigns look-up tables and continuously refreshes the combined image at up to 10 Hz. This approach allowed us to monitor the neuron morphology and the patch pipettes with a sufficiently high frame rate. We then targeted the fluorescence labeled axon bleb with a second recording pipette. Limiting fluorescence exposure times to short pulses of 50 to 100 ms allowed positioning the pipette on axon blebs without compromising neuron viability. Based on this approach we could successfully record from layer 2/3 pyramidal neuron soma and axon blebs at 128 ± 20 μm distance from the soma that showed a soma-to-axon delay of 140 ± 90 μs (*n* = 5, Figure 2C).

Another application of TEAMSTER is targeting of fluorescent protein expressing cells in transgenic animals. To identify fluorescent labeled cells, we used a foot-switch to activate an LED or a mechanical shutter in front of an arc lamp to limit fluorescent light to the duration of foot-switch push. We routinely used this approach to identify cyan fluorescent protein (CFP) expressing oligodendrocytes in a reporter mouse line (Battefeld et al., 2016, 2019). The example shows a CFP^+^ oligodendrocyte with a whole-cell recording pipette. The oligodendrocyte showed the typical hyperpolarized resting membrane potential and near passive responses to current injections (Figure 2D).

### POPSAR – Splitter for High-Speed Dual Imaging or Simultaneous Imaging and Photo-Activation

Optical recordings of the intracellular Ca^2+^ concentration ([Ca^2+^]_i_) are widely used to infer the underlying action potentials and resolve firing patterns of neurons. An advantage of epifluorescence-based imaging approaches is the possibility to image [Ca^2+^]_i_ in large fields of view with high (kHz) frame rates using CCD or sCMOS cameras. Combining Ca^2+^ imaging with activation of channelrhodopsins or calibrating [Ca^2+^]_i_ requires rapid switching between light sources to avoid cross-talk of imaging channels. To allow pseudo-simultaneous high-speed (up to 1 kHz) imaging with two excitation light sources precise control of LEDs is necessary. Based on a previously published approach (Miyazaki and Ross, 2015) we built an affordable stand-alone implementation that we called POPSAR to rapidly assign alternating frame exposure times to two LEDs (Figure 1D and Supplementary Figure 1B). This sub 100€ device (Supplementary Table 3) is based on the Propeller ASC^+^ board allowing execution of parallel processes for near real-time computation. This implementation achieves high frame rates as only camera frame exposure ouputs are detected and experimental flexibility as acquisition properties are software coded. We confirmed the functionality of the splitter in two applications that are of use for neuroscience research, first, the combination of Ca^2+^ imaging and photo-activation and second, calibration of [Ca^2+^]_i_ using fura-2.

**FIGURE 3:**
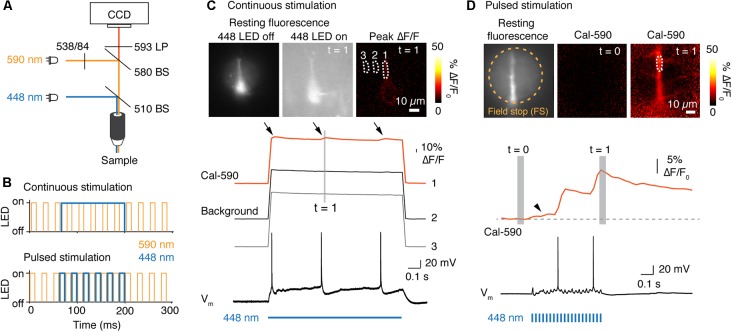
POPSAR microcontroller for simultaneous photo-activation of channelrhodopsin and Ca^2+^ imaging. **(A)** Light path schematic with used filters and beam splitters (BS) to achieve photo-activation and epifluorescence imaging. For photo-activation of the channelrhodopsin variant ChETA we used an LED with a center wavelength of 448 nm. Ca^2+^ was imaged with the red indicator Cal-590, excited with a 590 nm (peak) LED. **(B)** Two possible approaches for combining imaging and photo-activation. Photo-activation can either be achieved with continuous light (top) or with pulsed stimulation (bottom). **(C)** Neuronal Ca^2+^ was imaged at 40 Hz. To elicit a backpropagating action potential the ChETA expressing neuron was stimulated with a continuous pulse of light for 1 s. During photo-activation, total light intensity increased, resulting in a low signal to noise ratio of Cal-590 fluorescence. The three [Ca^2+^]_i_ peaks (arrows) correspond to the three action potentials (arrows). Continuous LED light leads to an increased total fluorescence and non-specific bleaching of the illuminated field of view. **(D)** Epifluorescence Ca^2+^ imaging at 40 Hz interleaved with photo-activation pulses for ChETA. Images of resting fluorescence, Cal-590 baseline signal and the [Ca^2+^]_i_ peak during the second action potential (top). The dendritic [Ca^2+^]_i_ dynamics of the selected ROI were plotted (middle) and aligned to the simultaneous electrical recording (bottom). Pulsed stimulation resulted in an increased signal-to-noise ratio and low background fluorescence in absence of stimulation due to red shifted imaging wavelengths. Note the slow increase of [Ca^2+^]_i_ during the first activation of ChETA (arrow head in [Ca^2+^]_i_ recording).

For combining Ca^2+^ imaging and photo-activation we used mice that express the channelrhodopsin variant ChETA (Gunaydin et al., 2010) specifically in layer 5 pyramidal neurons. Channelrhodopsin was activated with a blue LED (448 nm) and [Ca^2+^]_i_ imaged simultaneously with the synthetic Ca^2+^ indicator Cal-590 exited with a 590 nm LED (Figure 3A). We reasoned that a red-shifted Ca^2+^ indicator should avoid photo-activation of ChETA, which shows little residual activation beyond 575 nm (Gunaydin et al., 2010). We acquired Cal-590 fluorescence at 40 Hz with fluorescent light pulse durations of 10 ms that were initiated every 25 ms. We next tested whether photo-activation can be achieved with either continuous or pulsed stimulation of blue LEDs (Figure 3B). Continuous light (448 nm LED) for a 1 s channelrhodopsin activation period abruptly increased the resting fluorescence over the full field of view (Figure 3C). As a consequence, the signal-to-noise ratio decreased and we observed non-specific bleaching of the background over the full field of view (imaging traces Figure 3C). We could still detect light activated backpropagating action potentials, but the fluorescence change was only ∼3% ΔF/F [Ca^2+^]_i_ in the apical dendrite (Figure 3C). To improve the signal-to-noise ratio and reduce bleaching we examined whether interleaved light pulses for either imaging or photo-activation can be used. For a duration of 0.5 s, light was pulsed for each channel at a combined frequency of 80 Hz (10 ms light on) or 40 Hz/channel (Figure 3B). *Post hoc* separation of the two channels resulted in an excellent signal-to-noise ratio and a Ca^2+^ signal that was free of artifacts from photo-activation light. We observed peak [Ca^2+^]_i_ changes of ∼20% ΔF/F at the apical dendrite from light activated backpropagating action potentials (Figure 3D). Another advantage of a red Ca^2+^ indicator is that cellular structures located at greater depth from the surface can be imaged when compared to shorter wave length indicators. Simultaneous electrical recording confirmed that Ca^2+^ peaks were associated with single action potentials elicited by photo-stimulation (Figure 3D). Comparing these two approaches demonstrates that pseudo-simultaneous imaging and photo-activation results in improved Ca^2+^ signals while maintaining functional photo-activation at 40 Hz. Based on these results the latter mode was implemented into the POPSAR source code.

**FIGURE 4:**
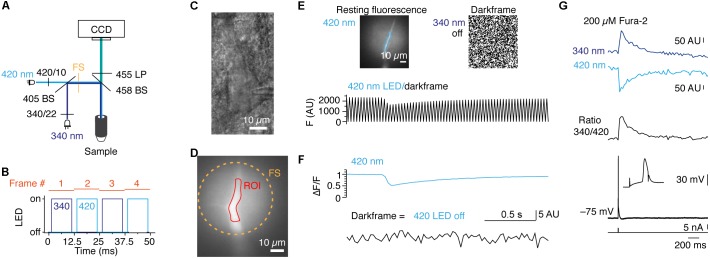
POPSAR and SLIDER microcontrollers for pseudo simultaneous ratiometric imaging with analog intensity control for calibration of ion concentrations. **(A)** Schematic of the microscope light path with LEDs, filters, and dichroic mirrors. The two excitation wavelengths were combined into a single light path in which optical elements were improved for UV transmission (see Methods). **(B)** Schematic of the timing of dual LED excitation. The 340 and 420 nm LEDs were switched on for 10 ms in an alternating manner and frames were initiated every 12.5 ms, resulting in an imaging frequency of 80 Hz. **(C)** Oblique illumination contrast image of a rat layer 5 neuron of the somatosensory cortex, recorded in whole-cell configuration and filled with 200 μM fura-2. **(D)** Fluorescence fura-2 image of the apical dendrite from the neuron in **(C)** from which fluorescence traces were extracted. **(E)** Images of 420 nm fluorescence and random noise pattern due to total darkness while the 340 nm LED was switched off. The partial illumination of the apical dendrite by the field stop can be seen and an ROI for signal extraction is indicated (top). Raw 420 nm fluorescence alternating with a dark frame results in a saw tooth pattern of the optical signal (bottom). **(F)** When the two channels are *post hoc* separated non-interfering fluorescence and dark frame signals are obtained. **(G)** Background subtracted fluorescence traces for 340 and 420 nm excitation (top) and 340/420 ratio of the two traces (middle). Voltage traces of corresponding single action potentials evoked by somatic current injection (bottom). The somatic action potential led to a backpropagating action potential into the apical dendrite and evoked [Ca^2+^]_i_ changes. Note that between the two excitation wavelengths the polarity of the fura-2 signals is reversed.

As a second application for POPSAR, we performed ratiometric imaging of fura-2 to, e.g., determine the absolute [Ca^2+^]_i_. Calibration of [Ca^2+^]_i_ necessitates to drive LEDs with reproducible and fixed current settings. In contrast to similar concepts that are provided in commercial systems which cost several thousand euros, SLIDER is an affordable add-on (∼100 €) as it is based on an Arduino compatible board and standard electronic components (Figure 1D and Supplementary Figure 1C and Supplementary Table 4). SLIDER is connected between POPSAR and the LED drivers allowing fine current adjustment (12 bit resolution) of the individual channels. When fura-2 was excited with two high-power LEDs with peak wavelengths of 340 and 420 nm we could collect the resulting emission with the same camera (Figure 4A). The addition of SLIDER introduced minimal delays (∼0.1 μs), which were negligible for an imaging frequency of 80 Hz and we confirmed functionality up to 1 kHz. At 80 Hz each frame was exposed for 10 ms and included an additional 2.5 ms buffer between frames to compensate for theoretical imperfect timing of LED on/off rise times and hardware induced delays (Figure 4B). For Ca^2+^ imaging, layer 5 rat pyramidal neurons were loaded with fura-2 during whole-cell patch-clamp recordings and the dye was allowed to diffuse into the neurons (Figures 4C,D). Subsequently, we imaged Ca^2+^ changes in the apical dendrite by evoking single action potentials with a somatic current injection. When one LED was switched off, every other frame recorded complete darkness resulting in a saw tooth pattern (Figure 4E). Precise timing and absence of crosstalk of the LEDs was confirmed after separating the two fluorescence channels (Figure 4F). Finally, we imaged Ca^2+^ with both LEDs resulting in [Ca^2+^]_i_ responses of opposite polarities allowing subsequent transformation to a ratio and alignment to the evoked action potential (Figure 4G). As short-wave UV light induces phototoxicity, we monitored resting membrane potentials of neurons during imaging recordings. The resting membrane potential was unaffected for 25 ± 8 min, before imaging: –64.7 ± 3.6 mV after imaging: –63.7 ± 4.4 mV (*n* = 5, *p* = 0.63, Wilcoxon signed Rank test). To summarize, we demonstrate the functionality of POPSAR for ratio-metric Ca^2+^ imaging and for combining Ca^2+^ imaging with photo-activation of channelrhodopsins allowing high-speed imaging of subcellular compartments.

## Discussion

In the present study, we describe the development of three microcontroller-based systems for brain slice illumination during patch-clamp recordings and epifluorescence imaging in combination with optogenetic activation. The low-cost devices are built around open-source hardware and free software and can be adapted to conventional microscopes. By using standard TTL commands the microcontrollers provide flexibility to connect a range of equipment and allow to keep existing components, e.g., external shutters. We created a GitHub repository that includes source code, part lists and detailed building instructions enabling the reproduction of the devices and rapid integration into existing microscope systems. If non-commercial LED illumination systems are adopted (Albeanu et al., 2008; Teikari et al., 2012; Bosse et al., 2015) an affordable (<500 €) and versatile microscope illumination system can be achieved for a fraction of the price of commercially available systems. At the same time the end-user remains in full control to adjust software settings to experimental needs and has the flexibility to add and exchange components.

Inspired by a system for rapid simultaneous imaging of two fluorescence probes (Miyazaki and Ross, 2015) we developed POPSAR and demonstrate its full functionality by simultaneous photo-stimulation and Ca^2+^ imaging. Similar LED triggering paradigms were recently implemented in head mounted miniscope solutions (Scott et al., 2018; Stamatakis et al., 2018). Stamatakis et al. (2018) utilized genetically encoded GCaMP6 and a red-shifted opsin. However, in this study channelrhodopsin activation was still observed while the LED for Ca^2+^ imaging was activated. In contrast, in our implementation we used the red-shifted synthetic Ca^2+^ indicator Cal-590 in combination with a blue LED to activate channelrhodopsin. By imaging pseudo-simultaneously and shifting the Ca^2+^ dye excitation into longer orange/red wavelengths we did not observe crosstalk of the two channels (Figure 3D). Moreover, POPSAR provides the opportunity to perform [Ca^2+^]_i_ calibration by using the ratiometric Ca^2+^ indicator fura-2. To achieve ratiometric fura-2 excitation we used a newly available high-power LED with a peak wavelength of 340 nm in combination with a 420 nm LED. Optimization of the optical path for UV light transmission and utilization of POPSAR and SLIDER for repeatable digital intensity control of LED current settings, revealed excellent performance for imaging frequencies up to 1 kHz. Implementing high-power UV-LEDs provides an efficient approach to investigate dynamic sub-cellular [Ca^2+^]_i_ changes in neurons or glial cells (Battefeld et al., 2019). The disadvantage of short wavelength (UV) light is that UV light induces phototoxicity more rapidly than longer wavelengths (Icha et al., 2017). The ability to accurately control and limit UV exposure times, as demonstrated here, was crucial for experiments using UV light.

The rapid LED imaging approaches are a useful tool to improve our understanding of neuronal assemblies, but also come with challenges associated with epifluorescence imaging. In contrast to two-photon imaging, single-photon excitation poses limitations in the control of the z-resolution as excitation light is not restricted outside the optical focus. Moreover, prolonged fluorescence exposure can lead to light induced damage (Icha et al., 2017). Precisely controlling exposure times by pulsing LEDs can thereby reduce overall fluorescence exposure and limit phototoxicity. Another approach of limiting excitation light in a sample is the recently developed wavefront shaping technique that makes use of computer-controlled spatial light modulators (SLM) to spatially restrict laser excitation light in tissue. Computer generated holography has the advantage of improving spatial specificity down to ∼10 μm in the axial direction thereby further reducing phototoxicity and increasing the signal-to-noise ratio (Foust et al., 2015). In future applications both laser based spatial control (SLM) and temporal control (TEAMSTER; POPSAR) could be combined to achieve pseudo-simultaneous recording of different indicators from diverse sub-cellular structures. Alternatively, POPSAR could be used for light-based activation of presynaptic terminals while recording optically Ca^2+^ or voltage in postsynaptic dendritic compartments to map the spatial distribution of inputs. These applications would significantly expand the toolset available for neuroscientists. Compared to two-photon microscopy the use of epifluorescence illumination increases light collection, has the cutting-edge advantage of achieving kilohertz acquisition rates, while allowing simultaneous imaging of a large field of view and detection of low intensity fluorescent signals (Jaafari et al., 2014). By changing excitation LEDs and filter combinations one could use a non-ratiometric Ca^2+^ reporter and structural dye to estimate Ca^2+^ concentrations (Delvendahl et al., 2015). In future imaging experiments one could combine two different synthetic Ca^2+^ probes (e.g., OGB-1 and Cal-590) in two neighboring cells thereby allowing dynamic imaging of cell-cell interactions. These applications are under the premise that optical crosstalk is non-existing and the appropriate filters are selected.

In summary, the microcontroller systems we presented here enable rapid and controlled LED excitation, expanding the toolbox for one-photon imaging and addressing new questions in the field of neuroscience.

## Author Contributions

AB, MK, and MP designed the research and edited the manuscript. MP wrote the scripts. AB, MP, and DvdW built and tested the hardware. AB acquired the data and drafted the manuscript. All authors contributed to the manuscript and approved the final version.

## Conflict of Interest Statement

The authors declare that the research was conducted in the absence of any commercial or financial relationships that could be construed as a potential conflict of interest.

## Footnotes

1 https://github.com/Kolelab/Image-analysis

2 PJRC.com

3 https://github.com/Kolelab/Fluorescence-Microscopy-Tools
