# Supplementary material for: A Versatile and Open-Source Rapid LED Switching System for One-Photon Imaging and Photo-Activation
Source: Front Cell Neurosci. 2019 Jan 17;12:530. doi: 10.3389/fncel.2018.00530 (PMC6344383; doi:10.3389/fncel.2018.00530)
Supplement: Supplementary file 1 [file Data_Sheet_1.PDF]

Supplementary Material

for

A versatile and open-source rapid LED switching system for one-photon imaging and photo-activation

Arne Battefeld, Marko A Popovic, Dirk van der Werf, Maarten H.P Kole

1.1 Supplementary Figures

Figure 1

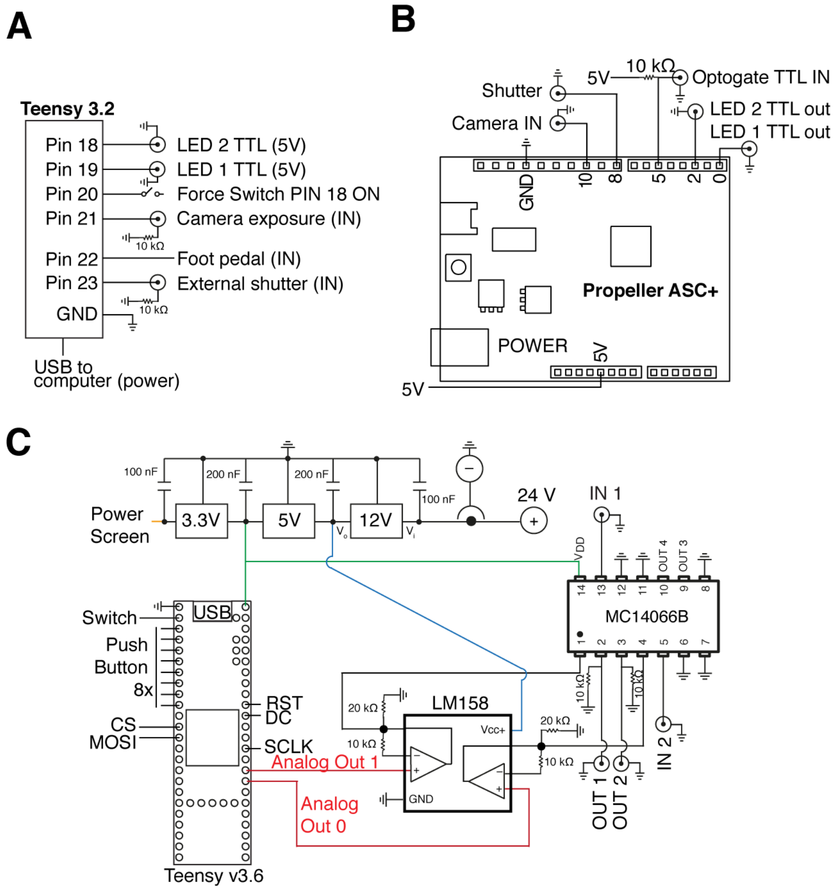

(**A**) Schematic wiring diagram for the Arduino based controller TEAMSTER, which is powered and interfaced via a USB connector for control in µManager. (**B**) Schematic wiring diagram of the high-speed splitter POPSAR depicting the Propeller ASC+ processing board with corresponding connections and pin layout. Power is provided by an external power supply (not shown). (**C**) Complete circuit diagram showing connections and components of SLIDER to control LED intensities. Three voltage regulators (12 V, 5 V and 3.3 V) supply the power for the various electronic components. For each output channel an independent operational amplifier converts the analogue output voltage (max. 3.3 V) of the Arduino Teensy to values between 0 to 5 V. The amplified voltage is fed into an analogue switch (MC14066B) that is gated by TTL input signals originating from POPSAR. The screen that provides visual feedback of selected LED intensities is connected and controlled by RST, DC, SCLK, CS, MOSI connections and powered by 3.3 V.

1.2 Supplementary Tables

Table 1: LEDs and drivers for transmitted light and epifluorescence illumination

Materials	Supplier	Catalogue #	Note	Cost (€)
LED 1 (e.g. 530 nm)	Thorlabs	M530L3-C1	Collimated LED. Can be replaced by other wavelength LEDs as needed or replaced by white light LED. For non-commercial cost-effective alternatives see (Bosse et al., 2015)	495
LED 2 (e.g. 730 nm)	Thorlabs	M730L4-C1	Near infra-red or far red if camera supports long wavelength. This choice of LED eliminates the need for IR filters and heat filters in the optical light path required by conventional lamps. For non-commercial cost-effective alternatives see (Bosse et al., 2015)	470
LED Drivers (2x)	Thorlabs LEDD1 or Cyclops LED driver (open-ephys)	LEDD1	We utilized the Thorlabs LED cube drivers and the more powerful cyclops driver that provides rapid current rise times. Driver receives TTL input as trigger signal.	ca. 280 or ca 450
BNC cable (4x)	RS-Components	426-2038	Generic 50Ω cables	10
Camera	Various (see methods)	N/A	Any brand possible as long as exposure can be triggered externally. CCD and sCMOS cameras were tested.	n/a

Table 2: List of materials for TEAMSTER

Item	Supplier/Manufacturer	Catalogue #	Note	Cost (€)
Teensy 3.2	PJRC.com	TEENSY32	Teensy 3.5 also possible. Other Arduino compatible board can be used as well. The current design requires a 5V tolerant board.	25
Footswitch	Farnell	1703843	Can be directly connected to the board or interfaced via any connector.	22
BNC connectors	Farnell	1020959	Panel mount	1.5/p
Switch	Farnell	2128119	Panel mount	3.5
Enclosure	3D printed	N/A	STL file available on GitHub repository	N/A

Table 3: List of Materials for POPSAR

Item	Supplier/Manufacturer	Catalogue #	Note	Cost (€)
Propeller ASC+	Parallax Inc.	32214	Can be purchased from RS components. Real-time processing board.	56
Power supply	Farnell	2451882	6-9 V power supply with 2.1 mm jack. The propeller board can also be powered via USB.	10
BNC connectors	Farnell	1020959	Any other panel mount BNC connector can be used	1.5/p
Enclosure	e.g. from Farnell	N/A	Use any enclosure box that fits the board.	Up to 30

Table 4: List of materials for SLIDER

Item	Supplier/Manufacturer	Catalogue #	Note	Cost (€)
Analogue Switch MC14066B	ON Semiconductor	MC14066B		0.5
Operational amplifier LM158	Texas Instruments	1459505 or 2474067	Other Op-Amp with similar specs	0.5
1.8" Color TFT LCD - ST7735R	Adafruit	358	3.3V-5V<br>160 x128 pixels	20
Teensy 3.6	PJRC	TEENSY36	3.3 V	30
Power connector	Farnell	2646488	Panel mount	4
Power supply	Farnell	1971798	Generic 24V	17
Voltage regulators (each 1x)	Farnell	12V: 2849721<br>5V: 1564483<br>3.3V: 1652296 or 1703357	12V, 5V, 3.3V<br>One might want to include heatsinks as the voltage regulators can get quite hot.	0.5<br>0.7<br>0.7
Switch	Farnell	9473602	Panel mount two way ON-ON switch	6
Push buttons (8x)	Farnell	2456210 or 2543089	Soldered onto breadboard	2
BNC connectors	Farnell	1020959	Panel mount	1.5/p
Capacitors (100 nF)	Farnell	9411887	Through hole	0.5
Resistors (4x 10 kΩ, 2x 20 kΩ)	Farnell	10 kΩ: 2329609<br>20 kΩ: 9342796	Through hole	1
Prototyping board	Farnell	1172108	Similar boards can be utilized and cut to size	6
Enclosure	3D Printed	N/A	Stl files provided on GitHub repository	N/A
